# PACO: a Shiny app for comparing perturbed pathways associated with different phenotypes

**DOI:** 10.1093/bioadv/vbaf212

**Published:** 2025-09-09

**Authors:** Giovanni Micale, Salvatore Alaimo, Alfredo Pulvirenti

**Affiliations:** Department of Clinical and Experimental Medicine, University of Catania, Catania, 95123, Italy; Department of Clinical and Experimental Medicine, University of Catania, Catania, 95123, Italy; Department of Clinical and Experimental Medicine, University of Catania, Catania, 95123, Italy

## Abstract

**Motivation:**

Pathways are biological networks describing interactions between genes, proteins, non-coding RNAs, drugs and chemical compounds that contribute to develop a specific metabolic function or biological process. Identifying perturbed pathways associated with a phenotype or condition helps to understand how functional processes are altered in complex diseases and which genes play a key role in these alterations. Recently, several algorithms have been developed to identify perturbed pathways associated with a phenotype. Still, no tools are available to visualize and compare perturbed pathways in the same species or different organisms.

**Results:**

Here, we present a web app called PAthway COmparator (PACO) to compare two or more sets of altered pathways associated with different phenotypes, starting from either custom data or simulation data returned by the pathway analysis algorithms MITHrIL and PHENSIM. The app allows users to visualize and compare the altered pathways, and zoom into specific regions. We show the potential applicability of PACO through a case study in which perturbed immune system pathways are compared in mice and humans after up-regulation of Interferon-stimulated gene 15 (ISG15).

**Availability and implementation:**

PACO is implemented as a Shiny R web application and is available at https://paco.dioncogen.eu/.

## 1 Introduction

Biological processes that regulate the cell’s activity are characterized by the expression, activation, or inhibition of several interrelated genes or proteins, which contribute to the realization of specific molecular functions. Such processes are called biological pathways, modeled as networks in which nodes are molecules involved in the process, and edges denote their physical or chemical interactions. Recent advances in high-throughput technologies have led to collecting several RNA and DNA sequencing data that helped better understand cells’ physiopathological status under specific conditions. In this context, identifying perturbed pathways associated with a specific phenotype is valuable for analyzing the alteration of biological processes in complex diseases. Metrics used to measure the degree of perturbation include the number of differentially expressed genes belonging to the pathway, the magnitude of their expression changes, and their interaction type, direction, and strength. Recently, several algorithms have been developed to identify perturbed pathways associated with a phenotype, such as Pathway-Express (Draghici *et al.* 2007), SPIA (Tarca *et al.* 2009), PARADIGM (Vaske *et al.* 2010), MITHrIL (Alaimo *et al.* 2016), and PHENSIM (Alaimo *et al.* 2021). These methods fully exploit the pathways’ topology to calculate perturbation scores for each pathway and each pathway node. All these tools start from gene expressions or log-fold changes of differentially expressed genes, except PHENSIM, which computes perturbation due to the up- or down-regulation of one or more molecules. In addition, several applications and web apps have been developed for pathway visualization and analysis. Pathview Bioconductor R package (Luo and Brouwer 2013) and KEGGScape Cytoscape plugin (Nishida *et al.* 2014) provide user-friendly interactive access, visualization, and exploration of pathways as hyperlinked graphs. Pathview Web server (Luo *et al.* 2017) extends the functionalities of Pathview, letting the user combine or compare multiple data types, samples, or experiments in a study using a graphical web interface. PathVisio (Kutmon *et al.* 2015) provides similar functionalities, where users can compare log-fold changes of gene expression in multiple conditions in pathways taken from WikiPathways (https://www.wikipathways.org/). ReactomeFIVIz (Wu *et al.* 2014) integrates visualization of pathways from Reactome (https://reactome.org/) with different types of statistical analysis (e.g. enrichment, gene set, probabilistic models). However, none of these tools is integrated with the aforementioned pathway perturbation analysis methods or allows the user to compare perturbed pathways associated with different phenotypes in distinct species. The latter could help highlight similarities or differences in the molecular mechanisms that regulate a specific biological process in different organisms. To fill this gap, we developed a Shiny R web app called PAthway COmparator (PACO). The app enables the visualization and comparison of two or more sets of altered pathways associated with different phenotypes in the same or different species, starting from lists of gene perturbation scores. Compared pathways are visualized as multilayer graphs that users can navigate by zooming into specific regions. With respect to existing tools, PACO does not focus on the integration of statistical analysis and visualization of pathways, but specifically aims to empower and generalize pathway-level phenotype comparison, in which a gene’s phenotype can be represented by any kind of metric (e.g. not necessarily its log-fold change of expression). PACO offers a valuable resource for biologists and physicians to better understand the functioning of biological processes and elucidate the role of genes in these processes.

## 2 Methods

In this Section, we illustrate the functioning of PACO app and how to use it. PACO documentation together with a step-by-step tutorial are available at https://github.com/knowmics-lab/PathwayComparator.

### 2.1 Data preparation

PACO app relies on pathway and homology data preliminarly downloaded and integrated from different sources. Currently, PACO supports three organisms (human, mouse, and rat), but more species will soon be integrated into the app. For each organism, we first collected all pathways present in KEGG Pathway (https://www.genome.jp/kegg/pathway.html) and Reactome databases. The pathways were then enriched by adding experimentally validated inhibitory interactions of miRNA targets with strong evidence, downloaded from miRTarBase (https://mirtarbase.cuhk.edu.cn/miRTarBase/miRTarBase_2025) and miRecords (http://miRecords.umn.edu/miRecords), and activation interactions of transcription factor (TF) -miRNAs obtained from TransmiR (http://www.cuilab.cn/transmir). Homology data include paralogs and orthologs so that the user can compare pathways of the same or different species. Paralogs have been downloaded from Paralog Explorer (https://www.flyrnai.org/tools/paralogs/web/). For each of the 3 organisms supported by PACO, a table containing all possible pairs of paralogous genes was built. Human orthologous genes in mice and rats have been extracted from MGI (https://www.informatics.jax.org/) and RatGenome (https://rgd.mcw.edu/) databases. For each pair of organisms supported by PACO, a table with all possible pairs of orthologous genes was built. Both pathway and homology data are updated monthly.

### 2.2 Structure of the app

**Figure 1.**
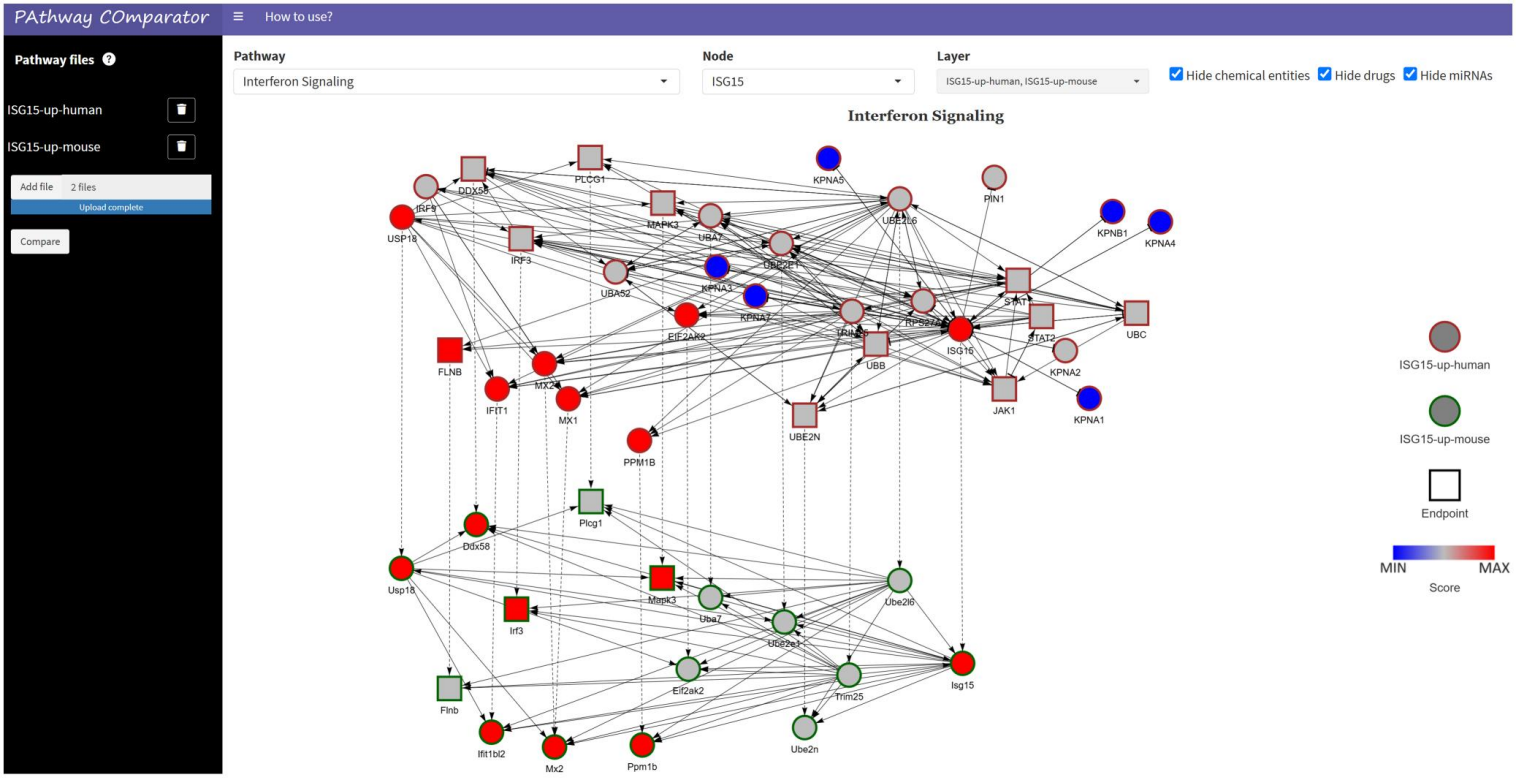
Structure of PACO app. Input data can be uploaded using the sidebar on the left and visualized in the main panel as a multilayer network, where each layer is a phenotype. In this example, we have two layers, representing the “Interferon Signaling” pathway in human (top layer) and mouse (bottom layer). Red nodes have positive perturbation scores, while blue nodes have negative scores. Nodes have different border colors, depending on the layers they belong to, and different shapes, depending on their role in the pathway (endpoint or not). Dashed edges connect homologous nodes. Visualization can be customized using filters on the top.

PACO has been implemented as a web app using the R Shiny framework (https://shiny.posit.co/). The web interface has two sections: a sidebar and a central panel (Fig. 1). The sidebar (on the left) can be used to upload two or more text files containing perturbation scores of single molecules (genes, miRNAs, or chemical compounds), associated to a specific phenotype in one of the species supported by the app. One file must be uploaded for each phenotype to compare and there is no limit in the size and the number of files that can be uploaded by the user. The central panel (on the right) is used to visualize altered pathways associated with the compared phenotypes, navigate through them, and customize the visualization.

### 2.3 Uploading input data

**Figure 2.**
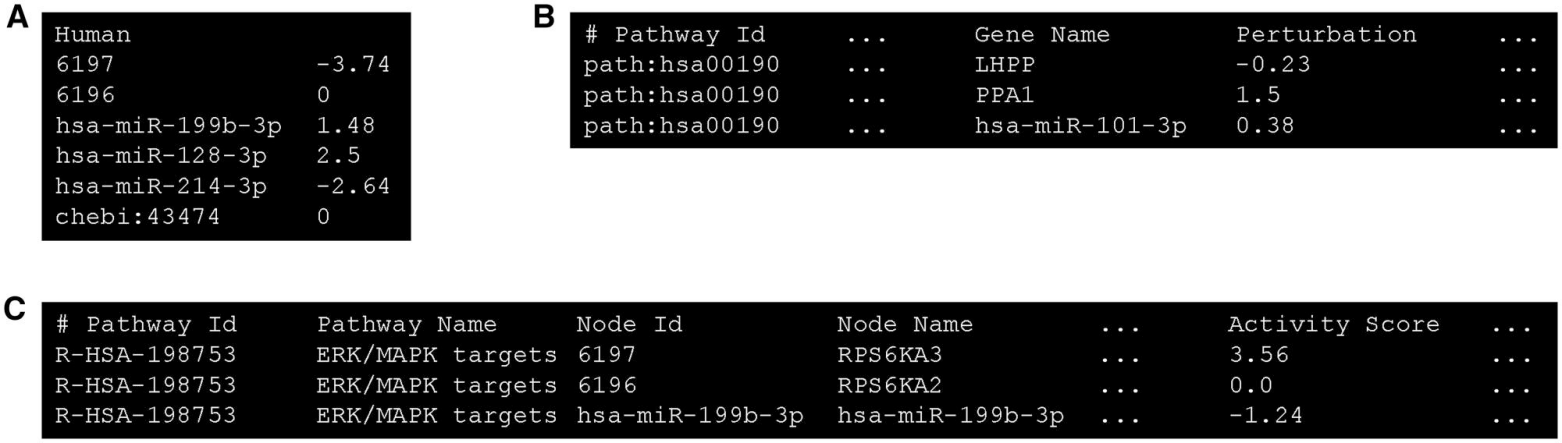
Formats of input data for PACO app: (A) A custom file, containing the organism to which molecules belong, followed by a list of their perturbation scores; (B) the perturbation file returned by MITHrIL algorithm, where scores are reported in the “Perturbation” column; (C) the simulation file returned by PHENSIM algorithm, where scores are reported in the “Activity Score” column.

By using the app sidebar, the user can upload a tab-separated two- columns text file containing a perturbation score for each biological element (Fig. 2A). The first line of the file specifies the common name of one of the organisms supported by the app (“Human,” “Mouse,” or “Rat”). The following lines indicate for each biological entity (gene, miRNAs, drug or chemical compound) the associated score. Genes are referred to by their Entrez IDs, miRNAs are identified by their entry name in miRBase (https://www.mirbase.org/), while chemical compounds and drugs are represented by their KEGG ids. The perturbation score can be any real number (positive, negative, or zero). Input files can refer to the same or different species, enabling comparisons within the same species or across different organisms. In addition, scores associated to biological molecules might not necessarily represent perturbations, but can be generic (e.g. log-fold changes or weights). To facilitate the integration of our app with the pathway analysis algorithms MITHrIL (Alaimo *et al.* 2016) and PHENSIM (Alaimo *et al.* 2021), the user can also upload a MITHrIL perturbation file (Fig. 2B) or a PHENSIM simulation file (Fig. 2C), which are tabular text files produced as output by the two algorithms, respectively. These files contain not only the perturbations of pathway nodes, but also several statistics about pathways and their nodes that are computed by the algorithms. In both cases, PACO extracts only the columns containing the ID of pathway nodes and their relative perturbations (“Gene Id” and “Perturbation” columns for MITHrIL, “Node Id” and “Activity Score” columns for PHENSIM).

### 2.4 Visualization

After uploading input data, by clicking on “Compare,” a multilayer network of pathways is shown, where each layer is a pathway associated with a specific phenotype (Fig. 1). Visualization makes use of visNetwork R package (https://datastorm-open.github.io/visNetwork/) and the Sugiyama layout. Intra-layer edges are depicted with solid lines, while inter-layer edges connect homologous genes and are represented by dashed lines. Nodes have different shapes, depending on their role in the pathway: square nodes represent endpoints (terminal nodes in the pathway that are known to affect the phenotype), while all other nodes have a circular shape. Pathway elements are filled in blue if they have negative scores, red if their score is positive, and grey if their score is 0. If compared phenotypes refer to the same organism, the same pathway is visualized in different layers and pathway nodes may be differently coloured depending on their perturbation score in the relative layer. Nodes belonging to different layers have different border colors. By passing the mouse over a node, its score is visualized. Visualization can be customized by choosing (i) the layers to be shown, (ii) which pathway (among those in common between the selected layers) should be compared, and (iii) whether miRNAs, chemical compounds and drugs should be visualized or not in the multilayer network. To avoid computational bottlenecks due to the visualization rendering and simplify the visualization, up to three layers at a time can be visually compared, and miRNAs, drugs, and compounds are not shown by default. The user can also select one of the molecules in one of the compared pathways. In this case, the visualization zooms into specific regions of the multilayer network, showing the selected molecule’s local neighborhoods (the ego networks) and its homologous nodes (if any). The latter functionality is handy when a pathway is large and challenging to explore visually.

## 3 Results

As a case study, we utilized PACO to compare the perturbed immune system pathways in mice and humans following the upregulation of Interferon-stimulated gene 15 (ISG15), a ubiquitin-like protein that functions both as an extracellular cytokine and an intracellular protein modifier. Recent research has focused on the role of ISG15 in SARS-CoV-2 and other viral infections, indicating that it has both anti- and pro-viral effects, acting as a post-translational modifier or a “cytokine-like” molecule during infection. We initially conducted two simulations in PHENSIM by upregulating ISG15 in humans and mice. Using the web portal of PHENSIM (https://phensim.tech/), we launched the simulations and downloaded the output simulation files. Then, we uploaded the two files into PACO and clicked on the “Compare” button. For the visualization, we chose the “Interferon Signaling” pathway, which is crucial for the immune response, and focused on the “ISG15” gene. The resulting multilayer network is illustrated in Fig. 1. It reveals several genes that are active in mice but not in humans (such as DDX58, EIF2AK2, and MAPK3) and a set of human importin genes (KPNA1, KPNA3, KPNA4, KPNA6, KPNA7, and KPNB1) that show reduced activity compared to normal conditions and lack orthologs in mice. Importin genes are integral to the formation of the nuclear pore complex, facilitating the selective transport of various molecules across the nuclear envelope. These findings suggest distinct immune system response behaviors between the two organisms, warranting further investigation.

## 4 Conclusion

In this paper, we presented PACO, a Shiny R web app for visualizing and comparing altered pathways associated with different phenotypes in the same or distinct species due to the perturbation of one or more biological molecules. PACO inputs a list of perturbation scores that can result from a user’s custom analysis or the output of pathway analysis methods MITHrIL and PHENSIM. The visualization can be customized by focusing on specific nodes to facilitate the exploration of compared altered pathways. As shown in the case study, PACO is not only a tool to compare different phenotypes, but also a valuable resource to compare species at the system biology level, helping to better understand the molecular mechanisms that entangle the functioning of complex biological processes and how the latter evolved during time. As future steps, we plan to (i) improve the visualization, (ii) support a higher number of species, (iii) provide better integration of our tool with pathway analysis algorithms, and (iv) extend the functionalities of PACO by including, e.g. enrichment analysis and network alignment methods.

## Data Availability

PACO is available at https://paco.dioncogen.eu/. Example data and documentation of the app are available at https://github.com/knowmics-lab/PathwayComparator.
